# Fructan synthesis, accumulation and polymer traits. II. Fructan pools in populations of perennial ryegrass (*Lolium perenne* L.) with variation for water-soluble carbohydrate and candidate genes were not correlated with biosynthetic activity and demonstrated constraints to polymer chain extension

**DOI:** 10.3389/fpls.2015.00864

**Published:** 2015-10-15

**Authors:** Joe A. Gallagher, Andrew J. Cairns, David Thomas, Emma Timms-Taravella, Kirsten Skøt, Adam Charlton, Peter Williams, Lesley B. Turner

**Affiliations:** ^1^Institute of Biological, Environmental and Rural Sciences, Aberystwyth UniversityGogerddan, Aberystwyth, UK; ^2^The Biocomposites Centre, Bangor UniversityBangor, UK; ^3^Department of Chemistry, Glyndŵr UniversityWrexham, UK

**Keywords:** biorefining, copy number variation, fructosyltransferase, genetic variation, polymer chain length

## Abstract

Differences have been shown between ryegrass and fescue within the *Festulolium* subline introgression family for fructan synthesis, metabolism, and polymer-size traits. It is well-established that there is considerable variation for water-soluble carbohydrate and fructan content within perennial ryegrass. However there is much still to be discovered about the fructan polymer pool in this species, especially in regard to its composition and regulation. It is postulated that similar considerable variation for polymer traits may exist, providing useful polymers for biorefining applications. Seasonal effects on fructan content together with fructan synthesis and polymer-size traits have been examined in diverse perennial ryegrass material comprising contrasting plants from a perennial ryegrass F2 mapping family and from populations produced by three rounds of phenotypic selection. Relationships with copy number variation in candidate genes have been investigated. There was little evidence of any variation in fructan metabolism across this diverse germplasm under these conditions that resulted in substantial differences in the complement of fructan polymers present in leaf tissue at high water-soluble carbohydrate concentrations. The importance of fructan synthesis during fructan accumulation was unclear as fructan content and polymer characteristics in intact plants during the growing season did not reflect the capacity for *de novo* synthesis. However, the retention of fructan in environmental conditions favoring high sink/low source demand may be an important component of the high sugar trait and the roles of breakdown and turnover are discussed.

## Introduction

Recently, the capacity for *de novo* fructan synthesis and seasonal variation in fructan content and polymer size were examined in a *Festulolium* monosomic substitution line family (Gallagher et al., 2015a). Significant differences between ryegrass and fescue for a number of traits were identified. Synthesis of polymers with a degree of polymerization (DP) greater than six sugar units appeared to be slow in the fescue plant examined and it had low polymeric fructan content and a high oligomeric/polymeric fructan ratio. Conversely, extension of polymer length from DP10/DP20 upwards probably occurred more freely, and, unlike ryegrass, fescue had a relatively even spread of polymer chain lengths above DP20. This included the presence of some very large polymers. Additionally fescue retained high concentrations of fructan (both polymeric and oligomeric) during conditions of high sink/low source demand.

A large proportion of agricultural land area is occupied by grassland in many temperate regions of the world, but relatively few forage grass species are cultivated. Perennial ryegrass (*Lolium perenne* L.) is the most widely grown species on non-marginal land (Burgon et al., 1997). Traditionally this has been used for livestock production and expertise exists within the agricultural industry to produce good herbage yields from modern varieties, but plant carbohydrates like the fructan polymers of temperate forage grasses are of increasing interest as renewable feedstocks to replace petrochemicals in the generation of fuels and the production of high value chemicals (Van Ree and Annevelink, 2007). There are “grass”-based biorefinery initiatives across Europe including in Ireland, Belgium, Austria, Poland, Germany, and the Netherlands producing materials such as organic acids, insulation products, high value chemicals including pharmaceuticals, alcohols, and animal feeds (Van Ree and Annevelink, 2007). To increase the commercial viability of a grass biorefinery, it is necessary to maximize the number of high value products that can be obtained from the process. Polymers and surfactants are widely used in a broad range of commercial sectors including the food, pharmaceutical, cosmetic, personal care and coatings industries. The global demand for surfactants was 19.2 million tonnes (including soap) in 2000 (Houseman, 2000). Carbohydrate surfactants are of interest because they are biodegradable and non-toxic to the environment (Warwel et al., 2001; Vieira de Almeida and Le Hyaric, 2005), and the demand for biosurfactants produced from renewable sources is likely to expand rapidly with increasing pressure to reduce the reliance on petroleum-derived products (European Union Advisory Group for Bio-based Products, 2009). If variation in fructan polymer traits similar to that in the *Festulolium* sublines was available within ryegrass then a similar range of polymers would be readily available for biorefining from current agricultural activities.

Considerable variation for fructan content has previously been shown within perennial ryegrass (Turner et al., 2006). It is this variation that has enabled the recurrent selection breeding programme at Aberystwyth to realize significant gain in water-soluble carbohydrate (WSC) content over the past 30 or so years (Humphreys, 1989; Wilkins and Humphreys, 2003), but little work has been carried out on the genetic control of polymer structure. The range of variation in the WSC content of the youngest fully expanded leaf in the spring was 87–286 mg/g dry matter (DM) in the IBERS WSC F2 mapping family (Turner et al., 2006). Total fructan varied from 7 to 231 mg/g DM. The data for total herbage from two perennial ryegrass populations phenotypically-selected for divergent WSC content were 20–250 mg/g DM WSC (Farrar et al., 2012) and 8–79 mg/g DM fructan (Gallagher et al., 2015b) also in spring. These populations therefore provide material with a similar range of WSC content to the *Festulolium* sublines studied previously when total herbage seasonal mean values were 109–163 mg/g DM WSC and 31–81 mg/g DM fructan (Gallagher et al., 2015a).

Perennial ryegrass is reported to accumulate fructan consisting of a mixed range of β(2,1) and β(2,6) molecules varying in chain length from 3 to ~90 units (Gallagher et al., 2007). The presence of the suite of fructosyltransferase enzymes required to synthesize these molecules has been confirmed in *Lolium* (Chalmers et al., 2005; Gallagher et al., 2007; Lasseur et al., 2011). Sucrose:sucrose 1-fructosyltransferase (1-SST) catalyzes the first reaction, the synthesis of the trisaccharide sugar 1-kestotriose from sucrose. The chain is further extended by addition of fructose units from sucrose by sucrose:fructan or fructan:fructan 6-fructosyltransferases (6-(S/F)FT) (FT2:2 of Gallagher et al., 2004) or by fructan:fructan 6G-fructosyltransferase (6G-FFT). 6G-FFT catalyzes the transfer of fructose from a fructan molecule to glucose (C6) of either a sucrose or a second fructan molecule forming fructans of the inulin neoseries type, but has also been shown to have fructan:fructan 1-fructosyltransferase (1-FFT) activity under some circumstances. Fructan 1-exohydrolase (1-FEH) hydrolyses β(2,1) linkages within fructan molecules but has previously been associated with high fructan-synthetic activity. It is possible that this enzyme has a role in fructan synthesis addition to the fructosyltransferases, as proposed by Lothier et al. (2007). TaMYB(myeloblastosis)13 transcription factors from wheat have recently been demonstrated to be activators of fructosyltransferase genes (Xue et al., 2011; Huynh et al., 2012); to regulate fructosyltransferases (McIntyre et al., 2012) and to regulate the fructan biosynthetic pathway leading to enhanced fructan accumulation (Kooiker et al., 2013).

Copy number variation (CNV) is thought to contribute significantly to natural variation in plants and to play an important role in plant evolution and adaptation (Zmienko et al., 2014). Copy number variants (CNVs) generally consist of large segments of DNA (above 1 kb) and may encompass one or more genes. They can contribute significantly to intra-specific genetic variation and often have phenotypic effects. Duplication of some genes of fructan metabolism has recently been discovered from studies with the IBERS bacterial artificial chromosome (BAC) library (Farrar et al., 2007). This library was created from a high sugar plant from the IBERS WSC F2 mapping family. Two BACs, both containing 1-SST and 6-(S/F)FT, have been sequenced. In an F2 mapping family gene duplication can be distinguished by the number of alleles present, as a maximum of two alleles is possible for any individual locus. Primers for 6-(S/F)FT have shown that one BAC is constant (two alleles indistinguishable with these primers) in the mapping family. The other varies as a dominant marker (unpublished data) and may be the gene mapped by Hisano et al. (2008) as 6-SFT (AB186920). It derives from Aurora, the high sugar parent of the WSC F2 perennial ryegrass mapping family. The genes on these BACS have high sequence homology (>95%), with most variation occurring within intron regions, and thus amplification of PCR products for three alleles indicates two copies of these genes are present. Rasmussen et al. (2014) described two isoforms of 6G-FFT (6G-FFT_1 from AB125218 and 6G-FFT_2 from AB288057) and reported that high fructan synthesis was associated with the expression of only one of these. Although there was considerable homology between these two isoforms they had sufficiently different genomic sequence to be distinguished with specific primers. Recently the genes for these two copies of 6G-FFT have been distinguished in IBERS plant material.

This study was carried out firstly to test the hypothesis that variation in fructan content within perennial ryegrass will be accompanied by differences in fructan synthesis and polymer size profile comparable with that found in *Festulolium* sublines, and that these effects will vary over the growing season. Candidate gene studies have been included to test a second hypothesis; that duplication of genes involved in fructan synthesis underlies the high fructan trait in the plant material included in this study.

## Materials and methods

### Plant material

Two sets of plant material, both with wide variation for WSC content but derived by different methods, were chosen for this study. One set was from the WSC F2 mapping family which has a well-characterized genetic basis (Turner et al., 2006); two “groups” of 12 plants from the extremes of the range of WSC content. The second set comprised the 30 plants from each of the second-generation selected “populations” (C2^s−^ and C2^s+^), with low and high WSC respectively, from the EU GRASP project (Farrar et al., 2012) which were derived purely by phenotypic selection. Subsets (three plants in each case) were identified within each of these four “populations”/“groups” at the low and high extremes of the variation (i.e., low-low, low-high, high-low, and high-high) during the course of this work, and these are described as “selections.” All plants were maintained in a frost-free, unlit glasshouse throughout the year in 13-cm diameter pots and renewed annually from a small group of tillers.

Single copies of each individual plant were randomly arranged along the glasshouse and moved after every harvest except the first. Material was sampled on six harvest dates (April 19, June 6, July 5, August 2, September 20, and November 1) over the 2011 growing season. Maximum and minimum daily temperature data from the on-site meteorological station are shown on Figure S1. The glasshouse was well ventilated every day to avoid high temperatures, and closed at night if low temperatures occurred. Reproductive state was characterized with a floral development score (1, vegetative; 2, stem elongating; 3, head just emerged; 4, mid-way emergence; 5, fully emerged; and 6, anthesis) and then any flowering heads were removed and discarded. Top growth was then removed back to a stubble height of 4 cm sampled during early afternoon. This material was immediately frozen in liquid nitrogen, stored at −80°C, freeze-dried and then chopped into 3–4 mm pieces prior to extraction for WSC. As the whole plant was cut back during sampling, the material taken at subsequent harvest dates consisted of totally new growth. Leaf growth and WSC content are virtually independent of any basal carbohydrate reserves by 6 days after defoliation (Morvan-Bertrand et al., 1999; Turner et al., 2001) so each date can be treated as an independent measurement.

*De novo* fructan synthesis from sucrose was examined in all the “selected” plants with triplicate inductions using the excised-leaf system (Cairns and Pollock, 1988; Pollock and Cairns, 1991). Plants were shaded to < 20% ambient irradiance for 7 days in the glasshouse. All fully-expanded green leaves were removed and stood in water in a closed box. Time zero samples were taken, wiped dry and immediately frozen in liquid nitrogen. Leaves for induction were transferred to 25 ml conical flasks containing 20 ml 200 mM sucrose. The flasks were arranged randomly within the replicate blocks in a growth cabinet at 20°C and 400 μmol/m^2^/s irradiance for 24 h. Twenty-four-hour samples were removed from the flasks, washed thoroughly in clean water, wiped dry and immediately frozen in liquid nitrogen. All samples were stored at −80°C, freeze-dried and then chopped prior to extraction for WSC.

### Carbohydrate analysis

Extraction and analysis of WSC for quantification purposes and for assessment of the relative distribution of polymers of different chain lengths by high performance anion exchange chromatography (HPAEC) followed Gallagher et al. (2015a). Polymer traits were analyzed for the induction experiment and for the July samples from the seasonal experiment when both sets of plant material showed a good range of fructan content.

### Candidate gene analysis

Four candidate genes of fructan synthesis and its (putative) regulation were analyzed. These were amplified from genomic DNA and the PCR products run on agarose gels unless specified otherwise. The number of PCR products per plant within the F2 mapping family with high sequence homology to the fructan metabolism genes under study was mostly used to distinguish the number of gene copies present. 1-SST [AY245431 (Chalmers et al., 2003) and AM407402 (JA Gallagher, unpublished database entry)] was amplified by two separate primer pairs: 1-SST BAC F8/lol 1-SST R4 primers and SpanFT1 F6/1-SST BAC R4 primers. Both primer pairs produced a second dominant band when gene duplication was present. 6-(S/F)FT (AM407403, FT2:2 of Gallagher et al., 2004) was analyzed with primers IA1-F and IA1-R and the PCR products run on 5% denaturing (sequencing) polyacrylamide gels. Duplication was detected as the presence of a second dominant band in addition to the main constant PCR product. 6G-FFT was amplified with two primer pairs. LES 1-SST BAC f7 and LES 1-SST BAC r14 amplify Lp6G-FFT_1 (Rasmussen et al., 2014) which is homologous to AB125218. These primers produced an additional band from a second copy of the gene in some GRASP population plants which was derived from the Dutch mapping family parent from the founder plants. LBTisopu6G-FFT F2.1 and LBTisopu6G-FFT R2 produced a dominant band when the Lp6G-FFT_2 (AB288057) described by Rasmussen et al. (2014) was present. MYB13 was amplified with LpMYB13-f2 and Lpmyb13-R2 primers. These gave a single band on agarose gels. The PCR products were cleaned up with microClean (Microzone) and sequenced with the forward primer on an ABI capillary sequencer. Polymorphism was characterized by a C/G single nucleotide polymorphism (SNP) at approximately 300 bp into the sequence. Further details of all primers and PCR conditions are given in Table S1. Images showing examples of the banding patterns of the PCR products on gels are given in Figure S2. An overall gene copy number score for all three fructan synthesis genes was calculated from summing scores for the number of copies present weighting a single copy as 1 and the duplication as 2. For example a plant with single copies of the three genes would score 3, and a plant with three duplications would score 6.

### Statistical analysis

Analyses of variance (ANOVA) to compare populations/groups and selections were carried out with the standard menu-driven procedures included in GenStat® for Windows®, Version 13.2 (Payne et al., 2010). Post-ANOVA multiple comparison tests were performed with Tukey. Correlations to examine relationships between traits across the full range of WSC content in all plants were calculated as the product moment correlation coefficient for pair-wise combinations. Linear and exponential regression curves of the form *y* = *a*+*b*(*r*^*x*^) were fitted for selected traits from this data. The maximum likelihood programme (MLP 3.08; Ross, 1987) was used for the other curve fitting procedures and to carry out parallel curve analysis. Pair-wise multiple comparisons are not possible with this software but selected individual pair-wise test were carried out with subsets of the data.

## Results

### Seasonal variation

Under glasshouse conditions, flowering in this plant material was concentrated in June and July with observable variation in heading date between individual plants. Flowering was more frequent in the GRASP populations than in the mapping family plants. However, there were no significant differences in the mean heading date score between the populations/groups or selections on any harvest date for the GRASP plants alone, the WSC mapping family plants alone or the full set of plant material, and flowering has not been further taken into account.

Two-way ANOVA showed a significant (*P* < 0.001) interaction between main plant material set (mapping family or GRASP) and date for all the sugars measured and so the data for the two main plant sets have been analyzed separately. Mean carbohydrate contents for all harvest dates are shown on Table 1 (also represented graphically on Figure S3). There was considerable variation between plants within each population/group. This resulted in overlap of the range of values measured for all carbohydrates on all harvest dates. Nevertheless the high population/group had more carbohydrates than the low, mostly significant at *P* < 0.001. WSC content was highest in April in the GRASP material and in July in the mapping family material. The peak in fructan content occurred somewhat later; in April/June for the GRASP material and in August in the mapping family material. The ratio of oligomeric fructan/polymeric fructan was mostly greater in the “low” plants. The population/group effect was significant (*P* < 0.01) for both sets of plant material, but the date and interaction effects were only significant (*P* < 0.01) for the GRASP populations.

**Table 1:** **Mean seasonal carbohydrate content (mg/g DM) for the extreme low and high groups from the WSC F2 mapping family (*n* = 12) and for the low and high GRASP C2 populations (*n* = 30)**.

**Date**		**Polymeric fructan**	**Oligomeric fructan**	**Total fructan**	**Ratio oligomeric/ polymeric**	**Disaccharide**	**Monosaccharide**	**Total WSC**
		**Mean (min–max)**	**Mean (min–max)**	**Mean (min–max)**	**Mean (min–max)**	**Mean (min–max)**	**Mean (min–max)**	**Mean (min–max)**
**WSC F2**	**Group**							
April	Low	12.81 (3.5–31.4) ab |A	5.28 (2.5–10.3) abc |AB	18.10 (6.7–40.9) ab |A	0.480 (0.301–0.962) ab |A	54.51 (34.7–71.9) cd |B	20.01 (13.8–28.9)abc|BC	92.61 (68.6–140.5) b |B
	High	36.89 (6.7–62.9) bcd	9.99 (4.5–14.7) de	46.88 (11.2–77.6) cd	0.346 (0.170–0.852) ab	64.33 (49.3–78.6) de	22.15 (14.2–31.5) bc	133.36 (87.9–164.2) cde
June	low	19.66 (6.0–36.0) abc |A	7.63 (2.5–12.8) abcd |BC	27.29 (8.5–48.8) abc |A	0.393 (0.300–0.464) ab |A	65.47 (42.9–91.6) de |C	20.86 (12.5–35.4) abc |C	113.63 (80.3–149.3) bcd |B
	High	46.71 (9.3–81.5) d	13.76 (3.8–20.1) ef	60.47 (13.1–98.8) d	0.317 (0.213–0.477) ab	72.58 (60.3–87.9) e	24.92 (15.2–72.6) c	157.97 (124.4–190.8) ef
July	Low	31.82 (2.7–82.8) abcd |B	13.59 (5.9–21.0) ef |C	45.41 (13.9–100.3)bcd|B	0.835 (0.211–4.615) b |A	56.67 (34.5–70.6) cd |BC	45.82 (36.4–56.6) d |E	147.90 (89.3–200.8) def |C
	High	77.74 (43.8–122.6) e	14.16 (9.3–19.4) ef	91.90 (54.2–137.1) e	0.207 (0.084–0.370) a	63.43 (49.8–91.5) de	40.15 (24.1–53.5) d	195.49 (146.1–251.6) g
August	Low	29.24 (2.7–73.7) abcd |B	9.72 (2.7–15.7) cde |C	38.96 (5.5–89.3) abcd |B	0.441 (0.211–1.005) ab |A	59.29 (46.9–77.9) de |BC	12.63 (6.8–20.3) ab |A	110.88 (68.4–143.0) bc |B
	High	84.86 (40.5–113.8) e	16.47 (11.2–20.1) f	101.34 (51.7–132.1) e	0.202 (0.147–0.278) a	60.18 (52.6–68.0) de	11.56 (8.2–17.2) a	173.08 (120.1–201.3) fg
September	Low	8.74 (2.2–27.1) a |A	3.16 (1.7–6.4) a |A	11.90 (4.2–33.5) a |A	0.494 (0.229–0.920) ab |A	22.89 (7.9–30.0) a |A	11.57 (6.8–21.5) a |AB	46.36 (20.6–74.4) a |A
	High	29.56 (3.9–54.0) abcd	6.15 (1.5–9.1) abcd	35.71 (5.4–61.7) abcd	0.254 (0.143–0.483) a	31.26 (9.8—-40.6) ab	17.70 (12.4–35.9) abc	84.68 (39.3–119.5) b
November	Low	16.42 (2.2–57.3) abc |A	4.91 (0.8–11.9) ab |A	21.33 (4.0–64.6) abc |A	0.498 (0.128–1.031) ab |A	44.29 (17.2–59.2) bc |B	27.22 (17.3–43.0) c |D	92.84 (38.6–144.9) b |B
	High	41.03 (4.9–72.5) cd	7.73 (2.7–10.9) bcd	48.77 (7.6–78.6) cd	0.241 (0.084–0.556) a	58.13 (42.3–68.4) cd	40.87 (30.8–50.0) d	147.78 (83.6–189.6) def
Probability	Group	*P* < 0.001	*P* < 0.001	*P* < 0.001	*P* < 0.001	*P* = 0.006	*P* = 0.010	*P* < 0.001
	Date	*P* < 0.001	*P* < 0.001	*P* < 0.001	*P* = 0.557	*P* < 0.001	*P* < 0.001	*P* < 0.001
	Group × date	*P* = 0.006	*P* = 0.015	*P* = 0.015	*P* = 0.190	*P* = 0.439	*P* < 0.001	*P* = 0.604
Interaction LSD (0.05)		15.15	2.67	16.95			5.98	
**GRASP**	**Population**							
March	Low	25.37 (3.6–59.2) ab |B	14.80 (3.4–28.6) d |E	40.16 (7.0–87.1) b |B	0.680 (0.337–1.127) e |D	25.90 (7.8–50.6) ab |B	32.63 (11.0–74.5) cde |E	98.70 (30.0–162.7) bc |B
	High	48.94 (26.4–83.2) cd	24.85 (13.9–42.7) f	73.80 (41.5–117.2) c	0.536 (0.272–0.876) de	35.02 (13.4–56.5) b	38.48 (14.2–61.1) e	147.29 (99.4–215.1) ef
April	Low	73.43 (6.9—-158.8) cf |D	10.26 (5.0–17.6) c |B	83.69 (16.1–174.5) cd |D	0.193 (0.090–1.347) ab |A	62.26 (39.0–161.6)defg|DE	27.97 (11.3–139.8)cd|CD	173.91 (92.5–317.6) gh |D
	High	118.52 (60.7–149.1) g	14.09 (6.8–20.1) d	132.60 (67.5–169.2) e	0.120 (0.094–0.139) a	54.08 (38.6–83.4) cde	29.30 (7.8–57.4) cde	215.98 (146.0–270.5) i
June	Low	76.03 (14.6–118.5) ef |D	14.94 (7.7–26.2) d |DE	90.97 (23.2–136.1) cd |D	0.218 (0.132–0.588) abc |AB	46.54 (32.0–118.7) c |C	16.18 (9.0–71.1) ab |B	153.70 (80.2–213.0) efg |D
	High	110.43 (75.6–146.0) g	18.95 (14.4–27.4) e	129.38 (89.9–167.7) e	0.172 (0.135–0.215) a	51.82 (35.4–81.2) cd	23.65 (11.7–41.5) bc	204.85 (159.4–256.2) i
July	Low	58.96 (6.4–148.3) de |C	14.62 (7.0–24.5) d |CD	73.57 (14.7–167.1) c |C	0.424 (0.114–1.709) cd |C	67.01 (49.8–101.0) fg |F	35.25 (14.8–52.7) de |DE	175.83 (112.5–252.1) gh |D
	High	78.59 (13.5–128.9) ef	15.75 (10.1–24.7) de	94.34 (31.2–144.8) cd	0.282 (0.105–1.307) abc	71.43 (44.3–101.3) a	32.82 (11.5–51.6) cde	198.59 (126.8–240.3) hi
August	Low	35.18 (1.8–93.6) bc |C	10.23 (2.5–20.7) c |BC	45.41 (4.3–114.2) b |BC	0.382 (0.194–1.327)bcd |BC	64.98 (47.9–84.7) efg |EF	8.29 (4.7–14.8) a |A	118.68 (72.8–174.7) cd |C
	High	80.90 (9.6–111) f	15.72 (4.6–21.7) de	96.62 (14.2–132.8) d	0.214 (0.141–0.477) ab	63.72 (48.3–85.4) efg	9.03 (3.4–17.2) a	169.36 (104.6–200.4) fg
September	Low	8.00 (1.1–34.6) a |A	3.38 (1.0–8.4) a |A	11.38 (2.7–43.0) a |A	0.694 (0.228–1.639) e |D	15.34 (7.2–24.7) a |A	10.55 (7.0–17.5) a |A	37.27 (17.4–73.7) a |A
	High	19.23 (4.5–54.8) ab	5.22 (2.0–11.4) ab	24.45 (7.7–66.2) ab	0.319 (0.181–0.696) abc	23.60 (14.3–34.7) a	10.64 (6.8–14.8) a	58.69 (32.3–111.4) a
November	Low	9.25 (1.1–29.7) a |AB	5.02 (1.4–10.2) ab |A	14.28 (2.5–39.9) a |A	0.702 (0.303–2.307) e |D	47.80 (30.4–70.4) c |CD	25.82 (16.8–33.0) c |C	87.90 (61.9–117.9) b |B
	High	35.47 (5.7–61.8) bc	8.12 (2.5–15.4) bc	43.59 (8.2–70.7) b	0.277 (0.129–0.524) abc	59.55 (40.2–79.5) def	29.86 (16.4–45.7) cde	133.00 (84.1–176.6) de
Probability	Pop	*P* < 0.001	*P* < 0.001	*P* < 0.001	*P* < 0.001	*P* < 0.001	*P* = 0.021	*P* < 0.001
	Date	*P* < 0.001	*P* < 0.001	*P* < 0.001	*P* < 0.001	*P* < 0.001	*P* < 0.001	*P* < 0.001
	Pop × date	*P* < 0.001	*P* < 0.001	*P* < 0.001	*P* < 0.001	*P* < 0.001	*P* = 0.157	*P* = 0.014
Interaction LSD (0.05)		11.70	2.09	12.94	0.1218	6.50		15.32

*The ranges of values are shown. Interaction means in columns within each of the two sets of plant material followed by the same letter are not significantly different at the 5% level according to post-hoc multiple pair-wise Tukey tests. Similarly uppercase letters after bars in columns indicate whether data for date top level means are significantly different at the 5% level (Tukey)*.

### Fructan synthesis and polymerization

Because of the variation within the material four sub-sets of three plants each were chosen from the main sets of plant material for more detailed analysis of fructan synthesis and fructan polymerization traits. These “selections” were at the low and high extremes of the variation described above (i.e., low-low, low-high, high-low, and high-high). The identification of plants from the low populations/groups for the low-low and low-high selections was straightforward, as the same plants were at the extremes of the ranges for the majority of sampling dates across the growing season. The plants chosen were always within the top or bottom 16% of the GRASP population or 33% of the mapping family group. Such a strict approach was not possible for the high-low and high-high selections from the high populations/groups as there was much more variation in which plants were at the ends of the ranges from month to month. It was also necessary to take into account the availability of leaf material for the plants concerned. The choice was based mainly on the summer months when WSC and fructan contents were high, and the selections were in the extreme 35% of the ranges for most of the growing season.

**Figure 1:**
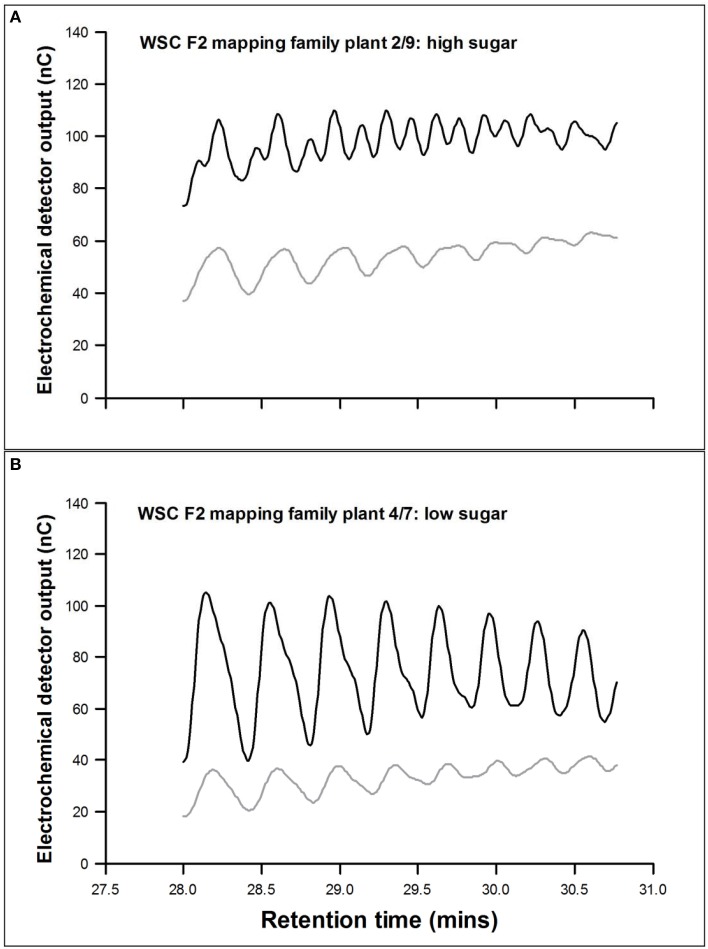
**Pattern of fructan polymers between DP28 and DP36 in two contrasting plants**. **(A)** WSC F2 2/9, a high sugar plant and **(B)** WSC F2 4/7, a low sugar plant. Black line, *de novo* fructan synthesis during induction; gray line, fructan present in July when fructan content is high.

The mean seasonal total WSC content of the mapping family selections was lower in the low selections than in the highs (Table 2), but fructan content (polymeric, oligomeric, and total) was only significantly lower (*P* < 0.05) in the low-low selection. The low-low selection also had a higher (*P* < 0.05) oligomer/polymer ratio. There were no significant differences for the major polymer above DP10, but the low-low selection had the smallest polymer-size range (*P* < 0.05), the lowest regression constant (*P* < 0.05), and the regression slope (*P* < 0.05). Peak doubling within a DP size due to the presence of isomers was sometimes observed. This was particularly obvious between 28 and 31 min (DP range 30–40) as shown by the sample sections of chromatogram in Figure 1. This peak doubling was scored on a scale of 0 to 4 where 0 = no isomer-doublet and 4 = isomer double peaks equal in height. Despite a range from a score of 1 in the lows to 3 or 4 in the highs these differences were not significant. During conditions of low photosynthetic activity retention of fructan (both polymers and oligomers) was much greater in the high selections (Table 2). There were no clear differences in fructan synthesis between the different plant selections. The GRASP selections showed very similar effects (Table 3). The low-low selection had lower WSC (*P* < 0.05), lower fructan (*P* < 0.05), and a higher oligomer/polymer ratio (*P* < 0.05) than the other three selections. It also had a lower incidence of isomer peak-doubling between DP30 and DP40. The high-high selection retained significantly more fructan during conditions where high carbon sink would be expected to outweigh low carbon supply. There were few significant differences in fructan synthesis between the different plant selections, but the high selections did produce larger fructan molecules than the low-low selection.

**Table 2:** **Fructan metabolism in the selections from the WSC F2 mapping family**.

**Dataset**	**Selection**	**Mean seasonal WSC**	**Fructan**				**Major polymer**	**Polymer profile–parameters for regression of peak height on chain length**		**Isomer doublet score**	**Largest polymer**
			**polymers**	**oligomers**	**total**	**oligomer/polymer ratio**	**>DP10**	**regression constant**	**regression slope**	**DP30–40**	
		**Mg/gDM**	**Mean seasonal fructan content mg/gDM**					**JULY–profile between DP50 and DP90/largest**			
Seasonal analyses	Low-low	87.7 a	11.7 a	5.7 a	17.5 a	0.713 a	41	3.21	−0.0731	1	99
	Low-high	119.7 ab	37.1 b	10.2 b	47.3 b	0.298 a	54	10.22	−0.2306	1	139
	High-low	152.7 bc	52.4 bc	11.6 b	64.0 bc	0.228 ab	52	11.60	−0.2738	3	136
	High-high	157.6 c	60.9 c	11.6 b	72.5 c	0.226 b	61	9.07	−0.1671	4	160
	Probability	*P* < 0.001	*P* < 0.001	*P* < 0.001	*P* < 0.001	*P* = 0.011	*P* = 0.265	*P* = 0.004	*P* = 0.005	*P* = 0.210	*P* = 0.024
	LSD (0.05)	26.65	15.87	2.95	18.01	0.3303		3.78	0.0923		35
			**Fructan content mg/gDM**								
Fructan retention	Low-low		4.7 ab	1.2 ab	5.9 ab	0.213 ab					
	Low-high		1.4 a	0.1 a	1.5 a	0.049 a					
	High-low		19.7 c	5.9 c	25.6 c	0.372 b					
	High-high		16.9 bc	5.4 bc	22.3 ab	0.185 ab					
	Probability		*P* = 0.002	*P* = 0.001	*P* = 0.002	*P* = 0.001					
	LSD (0.05)		10.31	3.23	13.30	0.1450					
			**Fructan content mg/gDM/24h**					**Profile between DP20 and DP70/largest**			
Fructan synthesis	Low-low		9.9 a	18.7 ab	28.5 a	2.000 a	19	3.60	−0.0963	2	83
	Low-high		19.1 a	23.8 b	42.9 a	1.440 a	20	3.97	−0.1081	2	77
	High-low		14.6 a	14.9 a	28.4 a	1.240 a	20	4.08	−0.0955	4	107
	High-high		10.7 a	18.8 ab	24.4 a	2.300 a	20	3.68	−0.0932	4	105
	Probability		*P* = 0.194	*P* = 0.038	*P* = 0.095	*P* = 0.308	*P* = 0.441	*P* = 0.871	*P* = 0.868	*P* = 0.453	*P* = 0.213
	LSD (0.05)			5.88							

*Fructan values for seasonal mean content and polymerization traits, retention after depletion of carbohydrates at low light and de novo synthesis during 24 h in the presence of exogenously supplied sucrose in the light. Probability and LSD estimates from One-way ANOVA for carbohydrate content: n = 3 plants per selection, plant values from triplicate assays. Means within each dataset in columns followed by the same letter are not significantly different at the 5% level according to post-hoc multiple pair-wise Tukey tests. Polymer traits (the major polymer >DP10, polymer profile between DP20 and DP50 and the largest observed polymer) are from single chromatograms after pooling the replicate extracts and regression parameters have been compared by parallel curve analysis with the maximum likelihood programme MLP 3.08*.

**Table 3:** **Fructan metabolism in the selections from the GRASP populations**.

**Dataset**	**Selection**	**Mean seasonal WSC**	**Fructan**				**Major polymer**	**Polymer profile–parameters for regression of peak height on chain length**		**Isomer doublet score**	**Largest polymer**
			**polymers**	**oligomers**	**total**	**oligomer/polymer ratio**	**>DP10**	**regression constant**	**regression slope**	**DP30–40**	
		**Mg/gDM**	**Mean seasonal fructan content mg/gDM**					**JULY–profile between DP50 and DP90/largest**			
Seasonal analyses	Low–low	101.7 a	11.6 a	6.2 a	17.8 a	0.858 b	14	1.37	−0.0307	2	61
	Low–high	150.2 ab	64.3 ab	13.9 b	78.3 b	0.317 a	42	11.15	−0.2530	4	136
	High–low	167.2 b	73.9 ab	14.2 b	88.2 b	0.236 a	58	11.69	−0.2354	3	141
	High–high	172.5 b	83.8 b	16.5 b	100.3 b	0.252 a	42	8.44	−0.1599	4	150
	Probability	*P* = 0.001	*P* < 0.001	*P* < 0.001	*P* < 0.001	*P* < 0.001	*P* = 0.144	*P* = 0.055	*P* = 0.152	*P* = 0.019	*P* < 0.001
	LSD (0.05)	37.17	22.06	3.98	23.96	0.1706				1	20
			**Fructan content mg/gDM**								
Fructan retention	Low–low		0.7 a	0.0 a	0.7 a	0.024 a					
	Low–high		2.0 ab	0.5 ab	2.5 ab	0.196 ab					
	High–low		2.2 ab	0.6 ab	2.8 ab	0.248 ab					
	High–high		6.5 b	3.4 b	9.8 b	0.339 b					
	Probability		*P* = 0.024	*P* = 0.021	*P* = 0.021	*P* = 0.031					
	LSD (0.05)		3.82	2.25	5.98	0.2078					
			**Fructan content mg/gDM/24 h**					**Profile between DP20 and DP70/largest**			
Fructan synthesis	Low–low		8.2 a	19.3 a	27.4 a	2.280 a	17	2.83	−0.0820	4	52
	Low–high		17.7 b	30.6 a	48.3 b	1.890 a	20	3.70	−0.1049	4	66
	High–low		18.5 b	26.0 a	44.5 ab	1.660 a	20	4.72	−0.1273	3	88
	High–high		13.4 ab	27.1 a	40.4 ab	2.180 a	20	3.03	−0.0809	4	88
	Probability		*P* = 0.011	*P* = 0.080	*P* = 0.016	*P* = 0.196	*P* = 0.306	*P* = 0.141	*P* = 0.200	*P* = 0.543	*P* = 0.036
	LSD (0.05)		6.50		13.04						27

*Fructan values for seasonal mean content and polymerization traits, retention after depletion of carbohydrates at low light and de novo synthesis during 24 h in the presence of exogenously supplied sucrose in the light. Probability and LSD estimates from One-way ANOVA for carbohydrate content: n = 3 plants per selection, plant values from triplicate assays. Means within each dataset in columns followed by the same letter are not significantly different at the 5% level according to post-hoc multiple pair-wise Tukey tests Polymer traits (the major polymer >DP10, polymer profile between DP20 and DP50 and the largest observed polymer) are from single chromatograms after pooling the replicate extracts and regression parameters have been compared by parallel curve analysis with the maximum likelihood programme MLP 3.08*.

### Relationships between polymer traits

Correlation analysis with the data from both plant sets was used to look at relationships between traits across the full range of variation in WSC content. The size of the major polymer, the size of the largest polymer (highest DP), the polymer profile constant and the extent of isomer peak-doubling were correlated (*P* < 0.05 or better) with fructan content in July (Table 4). Both polymer profile parameters and the extent of isomer peak-doubling were correlated with the size of the largest polymer. There were no correlations between *de novo* fructan synthesis and polymer traits of fructan extracted from plants in July. Furthermore, there were few correlations between polymer traits for the fructan produced during *de novo* synthesis (Table 5). The size of the major polymer did show a significant correlation (*P* < 0.05) with fructan content in July. In contrast with fructan from plants in July, isomer peak-doubling was negatively correlated with fructan content after 24 h *de novo* synthesis.

**Table 4:** **Correlation coefficients for polymeric fructan content (mg/g DM) and polymerization traits in all plants from the GRASP and the WSC F2 mapping family subsets combined in July when carbohydrate content was high**.

Content July	–						
Major DP	0.3457^*^	–					
Highest DP	0.7817^***^	0.5758^**^	–				
Profile constant	0.4451^*^	0.4012^*^	0.5873^**^	–			
Profile slope	−0.2804	−0.249	−0.4435^*^	−0.9678^***^	–		
Isomer peak doubling	0.5806^**^	0.2194	0.3560^*^	0.2918	−0.1743	–	
Induction synthesis	0.1415	0.0733	0.0481	0.2422	−0.2072	−0.1104	–
	Content	Major	Highest	Constant	Slope	Doubling	Induction

Fructan content produced by 24 h induction is also included n = 24. Significance threshold for

* P < 0.05 = 0.344, for

** P < 0.01 = 0.472, and for

*** *P < 0.001 = 0.599*.

**Table 5:** **Correlation coefficients for polymeric fructan content (mg/g DM) and polymerization traits in all plants from the GRASP and the WSC F2 mapping family subsets combined after induction for 24 h**.

Induction synthesis	–						
Major DP	0.0426	–					
Highest DP	0.0395	0.1806	–				
Profile constant	0.3162	0.1514	0.1285	–			
Profile slope	−0.3004	−0.0685	0.2531	−0.9088^***^	–		
Isomer peak doubling	−0.4006^*^	0.0853	0.0148	−0.1912	0.2009	–	
Content July	0.1415	0.4172^*^	0.1526	−0.1663	0.1601	0.241	–
	Induction	Major	Highest	Constant	Slope	Doubling	July

Fructan content in July when carbohydrate content was high is also included. n = 24. Significance threshold for

* *P < 0.05 = 0.344*,

^**^P < 0.01 = 0.472 and for

*** *P < 0.001 = 0.599*.

**Figure 2:**
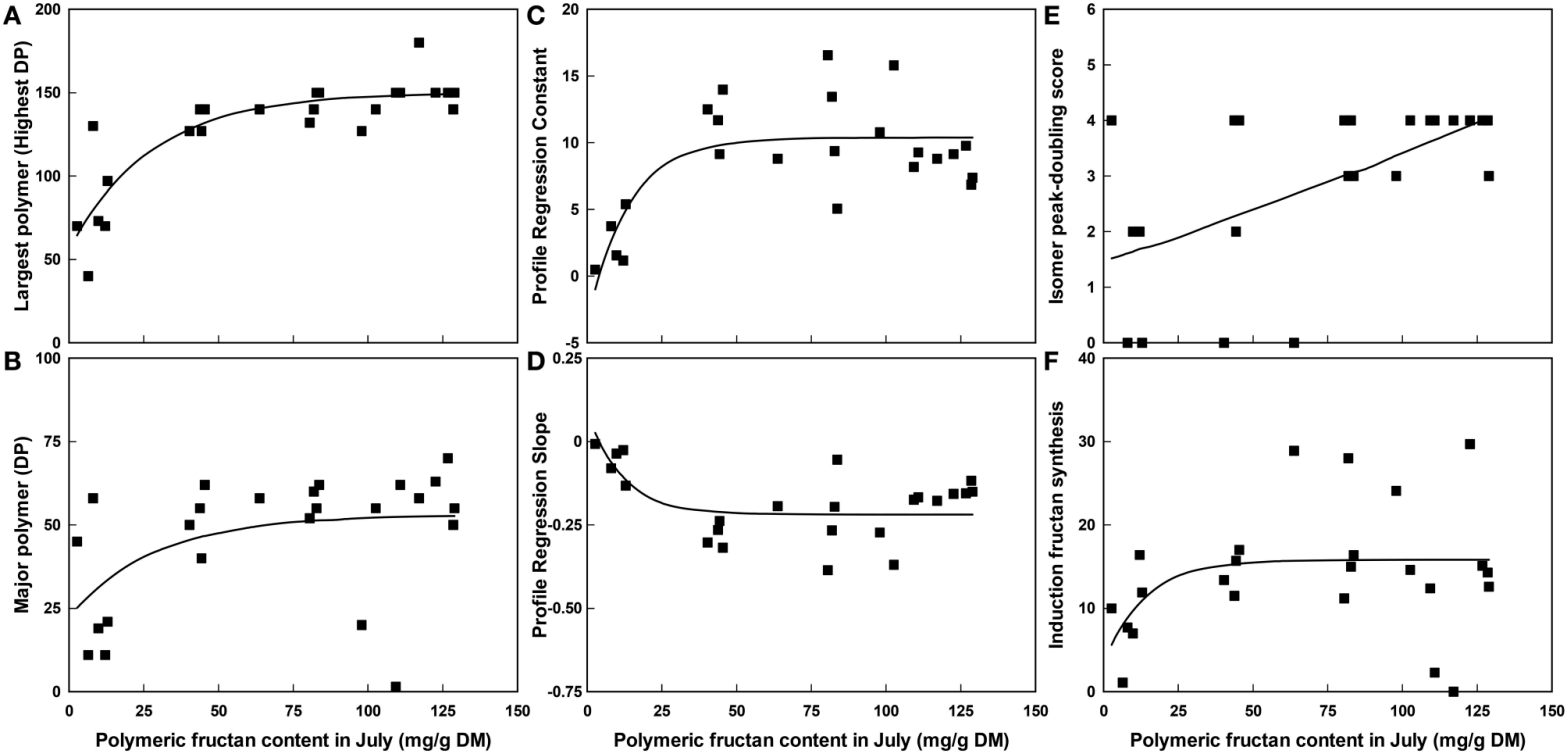
**The relationship between polymeric fructan content (mg/g DM) in July when carbohydrate content was high and selected polymer traits across all plants from the GRASP and the WSC F2 mapping family subsets combined, *n* = 24**. **(A)** The largest polymer present, **(B)** The major polymer present, **(C)** The constant of the profile regression, **(D)** The slope of the profile regression, **(E)** The isomer peak-doubling score and **(F)** Fructan synthesis during induction. Lines fitted by regression using an exponential curve.

The most significant of these relationships have been investigated further with regression analysis. An exponential curve [150.04-94.1(0.9646^x^)] was found to explain 73% of the variation (highly significant at *P* < 0.001) for the size of largest polymer present regressed on polymeric fructan content in July (Figure 2A). An exponential curve [53.08-30.7(0.9659^x^)] also best explained the variation for the size of the major polymer present regressed on polymeric fructan content in July (Figure 2B) but was not significant. The relationships of other polymer profile traits to polymeric fructan content were similarly explained by exponential curves (Figures 2C,D) with significant regression fits of *P* < 0.001 for profile regression constant [curve equation 10.389-13.76(0.9311^x^)] and *P* = 0.006 for profile regression slope [curve equation -0.2188+0.306(0.9201^x^)]. Together these showed that above a polymeric fructan content of 50–75 mg/g DM there were few observable further changes to the size of the polymers present in the polymeric fructan pool. In contrast the regression of isomer peak-doubling score on polymeric fructan content in July (Figure 2E) was near linear [−8+9(1.0019^*x*^)] and significant at *P* = 0.006 over the range of fructan contents measured. There was no significant regression of fructan synthesis in the leaf induction system on polymeric fructan content in July [curve equation 15.83-12.3(0.931^x^); Figure 2F].

### Fructan gene studies

Four genes of fructan synthesis and its (putative) regulation have been analyzed in the diverse plant material studied here (Table 6). A MYB13 gene has been included in addition to the 1-SST/6-(S/F)FT duplication found in the BAC library and the two 6G-FFT variants. 1-SST band duplication was detectable with two different primer sets depending on the source of the duplication. One of the observable duplications, visualized with the SpanFT1 F6/1-SST BAC R4 primer pair, was the one known from the BAC library which is present in the WSC F2 mapping family and therefore could occur in material from both the mapping family and GRASP population in this study. The other band duplication was new and was present in GRASP parent LTS05. This was visualized with the 1-SST BAC F8/lol 1-SST R4 primer pair and only occurred in the GRASP populations. Gene duplications were found in three of the selection-groups from the mapping family, and all the selection-groups from the GRASP populations. Three of the four duplications within the GRASP low populations were from GRASP LTS05 and only one was from the mapping family. The 6-(S/F)FT band duplication had also been found in the BAC library from the WSC F2 mapping family and therefore occurred in the mapping family and the GRASP plants in this study. Gene duplications were found in all selection-groups from the mapping family. Within the mapping family plants the 1-SST and 6-S/F)FT duplications tended to occur together. All the high-high selections from the GRASP plants contained the gene duplication, compared with only two plants from the remaining nine. As analyzed by the primers used here, a 6G-FFT type-1 gene duplication was present in GRASP parent LTS01. Three bands were present in the PCR products from this plant; two bands with a small indel difference and one smaller band. The two larger bands were of comparable size to the single band of the other LTS plants and these showed the highest sequence homology with 6G-FFT (the lower band of the gene duplication in LTS01 and the band from LTS18 both 99% with AF492836.2). BLAST searching also identified the extra third band as 6G-FFT, with closest homology to AF492836.2 (93%). This duplication only occurred in the GRASP population set in this study and was not common, occurring in only three of the plants studied here. All were high carbohydrate plants. The presence of a second copy of the 6G-FFT gene, the type-2 gene amplified with LBTisopu6G-FFT F2.1 and LBTisopu6G-FFT R2, was common in the material and derived from the low sugar parent of the mapping family, the *Lolium perenne* cultivar Perma. This occurred in all the selection groups from the mapping family, but not the high-high selections from the GRASP plants. MYB13, a recently described transcription factor with a putative role in the regulation of fructan metabolism in wheat, was also examined. One copy of this gene was detected with the primers used here. As analyzed by these primers, GRASP parent LTS01 has a double null allele leading to uncertainties in the full scores for some of the progeny. However, it is still apparent that the b allele is uncommon in the low sugar GRASP plants as “a.” represents “aa” or “a-null.” The same does not hold for the mapping family plants. The significance of gene duplication effects on fructan content in July and fructan polymer-profile traits was tested by ANOVA with the marker (D/S or aa/ab/bb) as a fixed effect (Table 7). Only one significant effect was detected; fructan content in July in the GRASP plants was affected by the 6G-FFT gene duplication. Mean fructan content for plants with one copy was 123.3 mg/g DM and for plants with a duplication 59.4 mg/g DM. Additionally a gene copy number score calculated from summing the gene copies present across all three fructan synthesis genes showed no correlation (correlation coefficient 0.0004) with polymeric fructan content in July across the full range of variation in WSC content. A linear regression line gave as good a fit as other curves, but was a horizontal line with 0.00 slope and a *P*-value of 0.998.

**Table 6:** **Genetic studies on selected candidate genes**.

**Set**	**Plant**	**Pop/Group**	**Selection**	**Gene**			
				**1-SST**	**6-(S/F)FT**	**6G-FFT**	**MYB13**
WSC F2	2/9	High	Highlow	S	S	D	ab
WSC F2	4/2	Low	Lowlow	D	D	S	aa
WSC F2	4/7	Low	Lowhigh	S	D	D	ab
WSC F2	6/4	High	Highhigh	D	D	D	ab
WSC F2	6/10	High	Highhigh	S	S	D	aa
WSC F2	7/10	High	Highhigh	S	S	D	bb
WSC F2	9/4	High	Highlow	S	S	S	aa
WSC F2	10/7	Low	Lowhigh	^*^	D	D	bb
WSC F2	15/5	High	Highlow	D	D	D	ab
WSC F2	16/4	Low	Lowhigh	S	S	D	ab
WSC F2	17/9	Low	Lowlow	S	S	D	bb
WSC F2	23/1	Low	Lowlow	D	D	D	ab
GRASP	C2 low 16	Low	Lowlow	D	S	D	null-null
GRASP	C2 low 29	Low	Lowhigh	D	D	D	a.
GRASP	C2 low 55	Low	Lowlow	S	D	D	a.
GRASP	C2 low 67	Low	Lowlow	S	S	D	a.
GRASP	C2 low 73	Low	Lowhigh	D^2^	S	D	b.
GRASP	C2 low 102	Low	Lowhigh	D	S	S	a.
GRASP	C2 high 353	High	Highhigh	S	D	S	b.
GRASP	C2 high 457	High	Highlow	S	S	D^2^	ab
GRASP	C2 high 462	High	Highhigh	S	D	S	b.
GRASP	C2 high 463	High	Highlow	D	S	D	ab
GRASP	C2 high 469	High	Highlow	D	S	D^2^	ab
GRASP	C2 high 474	High	Highhigh	D	D	S	a.

*The presence of duplications of genes of fructan metabolism is indicated as S, single copy; D, double copy; D^2^, double copy with both primer pairs used. Genotype scores for MYB13 are also shown*.

* *Missing value*.

**Table 7:** **Probabilities of significant effects (Genstat FPROB values) for fructan content and fructan polymer profile traits from one-way analysis of variance with marker score (D/S or aa/ab/bb) as a fixed effect**.

**Plant set**	**Trait**	**1-SST**	**6-(S/F)FT**	**6G-FFT**	**MYB13**
WSC F2 selections	Fructan content July	0.497	0.584	0.323	0.961
	Induction synthesis	0.183	0.163	0.863	0.481
	Fructan retention	0.495	0.256	0.312	0.783
	Polymer major DP	0.748	0.965	0.349	0.248
	Polymer highest DP	0.676	0.807	0.877	0.842
	Profile constant	0.058	0.080	0.879	0.916
	Profile slope	0.112	0.143	0.833	0.853
	Isomer peak doubling	0.872	0.602	0.789	0.945
GRASP selections	Fructan content July	0.439	0.360	0.015	0.311
	Induction synthesis	0.896	0.936	0.791	0.465
	Fructan retention	0.479	0.343	0.163	0.356
	Polymer major DP	0.189	0.966	0.620	0.364
	Polymer highest DP	0.390	0.462	0.101	0.298
	Profile constant	0.550	0.858	0.736	0.460
	Profile slope	0.577	0.864	0.530	0.645
	Isomer peak doubling	0.382	0.638	0.126	0.439
All selections	Fructan content July	0.684	0.750	0.174	0.495
	Induction synthesis	0.373	0.773	0.776	0.753
	Fructan retention	0.195	0.959	0.529	0.526
	Polymer major DP	0.519	0.916	0.596	0.132
	Polymer highest DP	0.757	0.525	0.187	0.224
	Profile constant	0.560	0.234	0.835	0.578
	Profile slope	0.614	0.305	0.627	0.660
	Isomer peak doubling	0.407	0.878	0.586	0.865

*WSC F2 selections, n = 12; GRASP selections, n = 12; all selections, n = 24*.

## Discussion

### Fructan metabolism

High WSC content preceded high fructan content during the growing season in this material as previously shown with *Festulolium* sublines (Gallagher et al., 2015a), despite the occurrence of a correlation between fructan and WSC content on many occasions in this as in other experiments (Turner et al., 2006). Within this study plants previously characterized as low carbohydrate plants generally had low fructan content and those characterized as high carbohydrate had high fructan content. However, there was great variability and near continuous variation in fructan content was observed in both plant sets. Although the WSC F2 low-low selections had lower concentrations of smaller fructan molecules with a more even spread of different polymer lengths, comparable with that observed for fescue (Gallagher et al., 2015a), overall there was little evidence of variations in fructan metabolism that resulted in any substantial differences in the size-complement of fructan polymers present in leaf tissue when fructan content was high. In general at lower ranges of polymeric fructan content, as fructan content increased, polymer chain length also increased. However, further chain extension did not appear to occur as fructan content increased above 50–75 mg/g DM. It therefore appears that new breeding programmes to introgress fescue traits will be necessary to produce cultivars with relatively higher concentrations of large fructan polymers for biorefining purposes. Peak doubling from the presence of isomers was correlated to high fructan content in July and did appear to continue to take place as the size of the polymeric fructan pool increased suggesting not only the activity of an additional enzyme but that this is under separate regulation. In contrast peak doubling was associated with reduced fructan synthesis. The advanced LC-MS methods described by Harrison et al. (2009, 2011) which give improved quantification and identification of individual isomers would provide further detail on these isomers. The wider importance of *de novo* synthesis for fructan accumulation *in vivo* was unclear. Fructan content and polymer characteristics in intact plants during the growing season did not reflect the capacity for *in vitro* fructan synthesis in leaves from the same plants. Together with significantly higher fructan retention under conditions of high sink demand in plants characterized as high accumulating plants, this raises the possibility that fructan breakdown and turnover may play a role in determining the fructan content of plants. Rasmussen et al. (2013) have modeled fructan synthesis with 1-SST and FT reactions and replicated polymer chain length distributions, but only for the range DP3–DP10. They also acknowledged that fructan breakdown and turnover could play an important role. The major enzymes of fructan catabolism in grasses are considered to be the fructan exohydrolases (FEHs) which release the terminal fructose from a fructan molecule. These are most closely related to cell wall invertases on phylogenetic dendrograms (Chalmers et al., 2005), and have been proposed to have putative roles in signaling and defense (Van den Ende et al., 2004). Lothier et al. (2007) cloned a 1-FEH from perennial ryegrass and showed that this exhibited high level of expressions under conditions of active fructan synthesis. They mapped this to a distal position on linkage group 3 (= chromosome 3). No QTL for WSC or fructan content were identified on chromosome 3 by Turner et al. (2006) but Cogan et al. (2005) did report WSC QTL on this chromosome in perennial ryegrass. More recent work has also implicated chromosome 3 as a likely candidate for the location of a major QTL for WSC/fructan content in the ryegrass genome. LOSITAN analysis for regions of the genome under significant selection pressure can indicate the existence of, as yet unknown, QTLs. This recently identified the SSR marker 14 ga1 on chromosome 3 as showing a significant effect during selection for WSC (Gallagher et al., 2015b). Additionally, it appeared that major genes involved in the control of at least some of the distinctive fructan-polymer-traits of fescue described by Gallagher et al. (2015a) might be located on fescue chromosome 3.

### Copy number variation

Gene duplications and their subsequent divergence can play an important role in the evolution of novel gene functions (Innan and Kondrashov, 2010). It has been proposed that the multiple copies of invertase genes and the variants of fructosyltransferase genes in perennial ryegrass arose during evolution from a common cereal ancestor invertase by gene duplication and rearrangement (Francki et al., 2006) and such genes can be clustered within the genome (Huynh et al., 2012). The recent discoveries of further duplication of fructosyltransferase genes would therefore be excellent candidates for a major role in regulating fructan and WSC content.

The two analyzed 1-SST banding patterns could be looking at the same gene duplication, but this is not necessarily the case. One plant, GRASP C2 low 73, was scored as showing both 1-SST duplications, and whilst this could result from scoring errors it does suggest that the duplications may indeed be different. It is therefore possible that this plant contains three copies of 1-SST. As the duplications of 1-SST and 6-(S/F)FT arising from the perennial ryegrass cultivar Aurora result from duplication of a piece of chromosome containing both genes within relatively short lengths of DNA as represented by the duplicate BACs, it is not surprising that these duplications tended to occur together in the WSC F2 mapping family. However, although this could again result from scoring errors, there is some evidence for recombination within one of the BACs in the WSC F2 mapping family as plant 4/7 has the 6-(S/F)FT duplication, but not the 1-SST duplication. After two rounds of recombination in the GRASP plants there is further evidence for recombination between the two genes. Four progenies (two high and two low) have the 6-(S/F)FT duplication but not the 1-SST duplication, and four (also two high and two low) have the 1-SST duplication but not the 6-(S/F)FT duplication. This suggests that there is little selection pressure to retain the duplication of the two genes together, even during phenotypic selection for WSC content.

Recently Rasmussen et al. (2014) have reported that high fructan synthesis was significantly correlated with the expression of only one of two isoforms of 6G-FFT, the 6G-FFT_2 form. The two isoforms represented two genes with relatively high sequence homology but distinctive polymorphism in some regions of the genomic sequence. 6G-FFT_1 was present in nearly all the plants examined, but was not positively correlated with fructan. High expression of 6G-FFT_2 was strongly correlated with polymeric fructan content, but was present in only some individuals. 6G-FFT_1 was present in all the plants included in this study, in agreement with the findings of Rasmussen et al. (2014). Furthermore a duplication of this gene was present in the plant material, although it was rare and only occurred in three plants. These were all from the low end of the high WSC selections; the duplication did not occur in any low WSC plants. 6G-FFT_2 was also present in most of the plants examined here; in 17 of the 24 individuals but with no clear relationship to fructan content. Two plants (GRASP C2 high 457 and 469) had both duplications. Although, “highs” these were toward the lower WSC end of the “high” range. However, Rasmussen et al. (2014) also report allelic variants, which cannot be distinguished with current primers, in their material and these showed uneven distribution across cultivars with different WSC content. One variant of 6G-FFT_2 occurred only in the cultivar Fennema which has low WSC content. The 6G-FFT_2 in the material studied here arose as a single allele from Perma, a cultivar with low/normal WSC content, and so may be a similar ‘low WSC’ allelic form. 6G-FFT _1 has been mapped to chromosome 3 (Hisano et al., 2008) which may contain regions regulating the WSC trait as discussed above. It could be carried by linkage even if not actively involved in determining the WSC trait.

Two FEH genes have been isolated from perennial ryegrass. A 1-FEH (DQ016297) has been mapped to chromosome 3 (Lothier et al., 2007). More recently, a 6-FEH (EU219846) has been described (Lothier et al., 2014). The 1-FEH did not amplify well in the IBERS WSC F2 perennial ryegrass mapping family and has not been included in the study, particularly as there does not appear to be any evidence of gene duplication. From the scores available for the plants used here, it is possible to determine that the low plants were all heterozygous or one of the homozygous genotypes. The only example of the other homozygous genotype was a highhigh individual. Work is ongoing on 6-FEH loci with homology to EU219846 where a gene duplication may be present.

A MYB13 gene was analyzed in addition to the other genes although no duplication has been identified, but there was no observable differentiation in genotype between the different groups. Indeed, overall in this study, there were no clear relationships between gene duplications *per se* or this MYB13 genotype and fructan content or capacity for *de novo* synthesis in the WSC F2 mapping family. The situation was not much different for the GRASP plant material, so there would seem to be little value in carrying out a wider study to properly test statistical significance. These results do not preclude the presence of other transcription factors (including other MYB genes) in the ryegrass genome, which do have regulatory functions for fructan biosynthesis. A recent study using association mapping has not yielded any better evidence as the candidate genes of fructan metabolism were most often correlated with fiber traits and carbohydrate traits were correlated genes from other areas of metabolism (Pembleton et al., 2013).

Finally, however, it should be borne in mind that the presence of a gene does not equate to high expression and transcriptional regulation may be crucial, particularly in relation to environmental variation. Rasmussen et al. (2014) clearly showed that it was the expression of a specific isoform that varied between plants under some conditions, even though it was present in the genome of both high and low fructan-accumulating individuals. Differential expression of the various gene copies in addition to that of allelic variants, would add a further layer of complexity to the regulation of fructan metabolism. These relationships need to be disentangled to fully understand the roles of biosynthetic and breakdown/turnover processes in determining the size and nature of the fructan pool in ryegrass plants.

## Conclusions

In conclusion, neither of the experimental hypotheses was proved in the form postulated. Rather an exponential relationship between polymer size and fructan content was demonstrated indicating an apparent restriction to polymer extension above chain lengths of around DP140 in perennial ryegrass. There was no clear relationship between gene duplication *per se* and polymeric fructan content. Further study of the candidate genes of fructan metabolism and their transcriptional regulation is required to fully elucidate the role of copy number variation. However, this is complicated by the considerable care involved in designing distinctive primers because of the high sequence homology present especially in coding regions. Mapping of the relationships to WSC QTL including epistatic QTL would then provide more information on the roles of biosynthetic and catabolic genes in the regulation of fructan metabolism in perennial ryegrass.

## Author contributions

JG, AC, and PW conceived and designed the study. AC, DT, ET, KS, and LT carried out the experiments and analyzed the data. JG and LT wrote the paper.

### Conflict of interest statement

The authors declare that the research was conducted in the absence of any commercial or financial relationships that could be construed as a potential conflict of interest.

## Abbreviations

ANOVA: analysis of variance
BAC: bacterial artificial chromosome
CNV(s): copy number variation (variants)
DM: dry matter
DP: degree of polymerization
FEH: fructan exohydrolase
1-FFT: fructan:fructan 1- fructosyltransferase
6-(S/F)FT: sucrose:fructan or fructan:fructan 6- fructosyltransferase
6G-FFT: fructan:fructan 6G-fructosyltransferase
HPAEC: high performance anion exchange chromatography
LSD: least significant differences
SNP: single nucleotide polymorphism
1-SST: sucrose: sucrose 1-fructosyltransferase
WSC: water-soluble carbohydrate.
