# Supplementary material for: Fructan synthesis, accumulation and polymer traits. II. Fructan pools in populations of perennial ryegrass (Lolium perenne L.) with variation for water-soluble carbohydrate and candidate genes were not correlated with biosynthetic activity and demonstrated constraints to polymer chain extension
Source: Front Plant Sci. 2015 Oct 15;6:864. doi: 10.3389/fpls.2015.00864 (PMC4606054; doi:10.3389/fpls.2015.00864)

Supporting information

Table S1. Primer information, PCR and analysis details for candidate gene analysis.

Gene	Primer	Primer Name	Primer Sequence	Annealing Temperature (°C)	PCR Cycles	Agarose gel %
1-SST	Forward	1-SST BAC F8	5’-GCAACACCGACACCCTAGC	62	35	1.0
	Reverse	lol 1-SST R4	5’-GATGAGGTTTGTGCGGGTCTTC			
1-SST	Forward	SpanFT1 F6	5’-GGGTTCCCGTGGAGCAAC	55	35	2.0
	Reverse	1-SST BAC R4	5’-CTAGGGTGTCGGTGTTGCCG			
6-(S/F)FT	Forward	IA1-F	5’- CGGAGGAGTTGTACGTGATAAAGC	65	35	n/a
	Reverse	IA1-R	5’- CTAGTGCCACTGTCCTCGGAATCGA			
6G-FFT	Forward	LES 1-SST BAC f7	5’-ATCGGCCTCAGGTACGACTG	55	35	1.0
	Reverse	LES 1-SST BAC r14	5’-GCTTCAACGGTGATGCCAC			
6G-FFT	Forward	LBTisopu6G-FFT F2.1	5’-ATGGAGATCCCGAGGAGC	64	35	1.0
	Reverse	LBTisopu6G-FFT R2	5’-GCCATAGGGGTTGTGCTGG			
MYB13	Forward	LpMYB13-f2	5’-ACGGAGCAGGAGGACCTGAA	60	35	1.0
	Reverse	Lpmyb13-R2	5’-GCATTGGTCGTCGTTGTTGT			

Figure S1 Meteorological data. Maximum (solid line) and minimum (dashed line) daily temperature (^oC) over the growing season from the Institute on-site meteorological station.

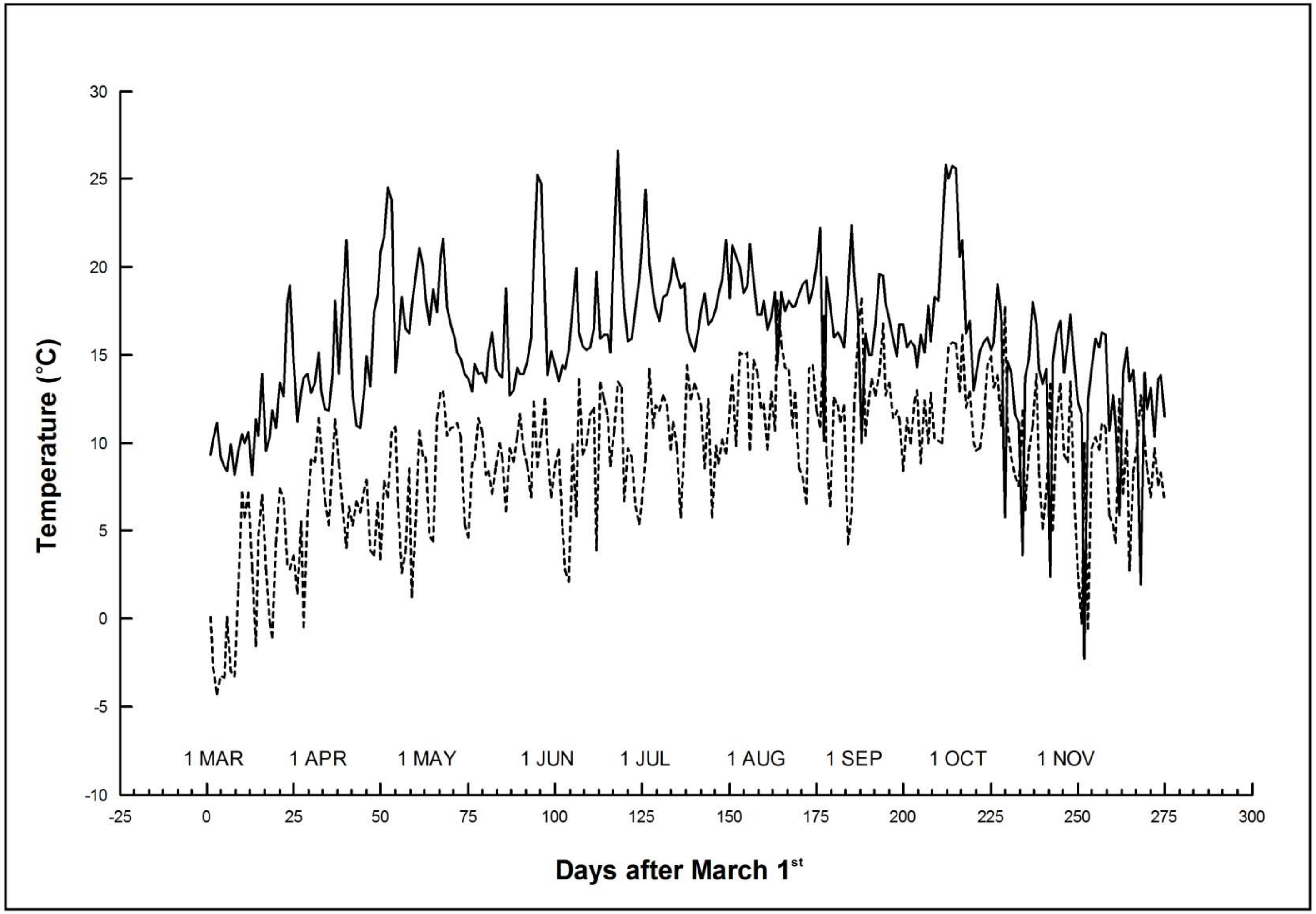

Figure S2. Images of banding patterns on gels for PCR products from A. Primers 1-SST BAC F8 and lol 1-SST R4 for 1-SST, B. Primers SpanFT1 F6 and 1-SST BAC R4 for 1-SST, C. Primers IA1-F and IA1-R for 6-(S/F)FT, D. Primers LES 1-SST BAC f7 and LES 1-SST BAC r14 for 6G-FFT and E. Primers LBTisopu6G-FFT F2.1 and LBTisopu6G-FFT R2 for 6G-FFT.

A. Example of banding pattern for primers 1-SST BAC F8 and lol 1-SST R4 (for 1-SST) in the GRASP population founder plants (negative image of gel)

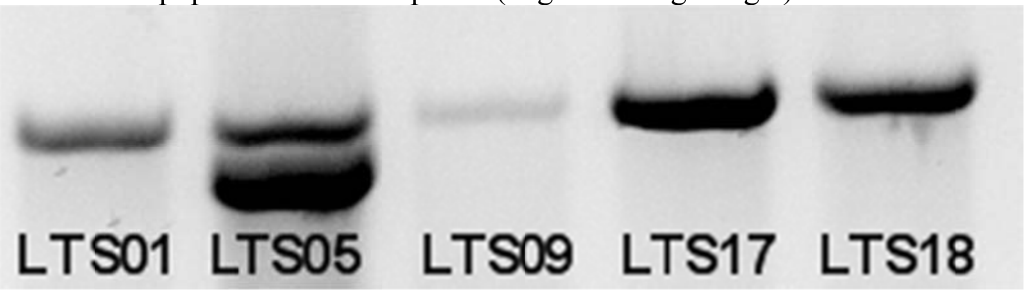

The lower band is only present in the founder plant in the second lane. The upper band of ≈1,500bp is present in all founders and all progenies (failed PCR in lane 3), and two polymorphic alleles (SNPs) are present in many cases including in the founder plant in lane 2. The presence of three alleles in a diploid plant shows two (duplicate) loci are present.

B. Example of banding pattern for primers SpanFT1 F6 and 1-SST BAC R4 (for 1-SST) in F2 mapping family plants (negative image of gel)

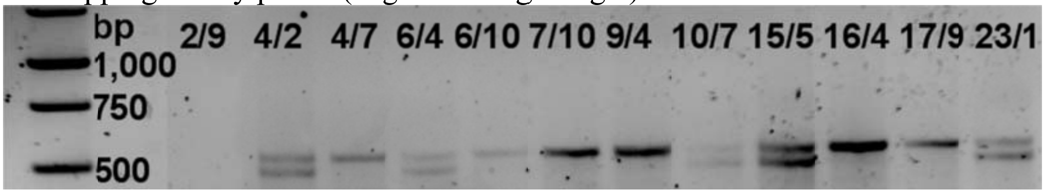

The 1-SST locus shown by the top band is present in all plants and represents two alleles. Failed PCR in lane 1, lane 8 (labelled 10/7) was not the correct DNA. The bottom band (in lanes 2, 4, 9 and 12) is dominant and represents a duplicate locus.

C. Example of banding pattern for primers IA1F and IA1R (for 6-(S/F)FT) in F2 mapping family plants

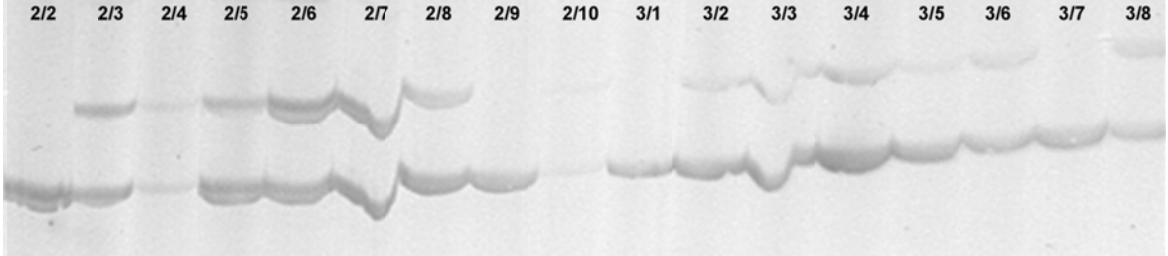

Bottom band (≈600bp) is 6-(S/F)FT gene 1 present in all plants (failed PCR in lanes 3 and 9) Top band (≈650bp) is 6-(S/F)FT second copy present in lanes 2, 4, 5, 6, 7, 11, 12, 13, 14, 15, and 17 and absent in lanes 1, 8, 10 and 16

D. Example of banding pattern for primers LES 1-SST BAC f7 and LES 1-SST BAC r14 (specific for 6G-FFT_1) in the GRASP population founder plants (negative image of gel)

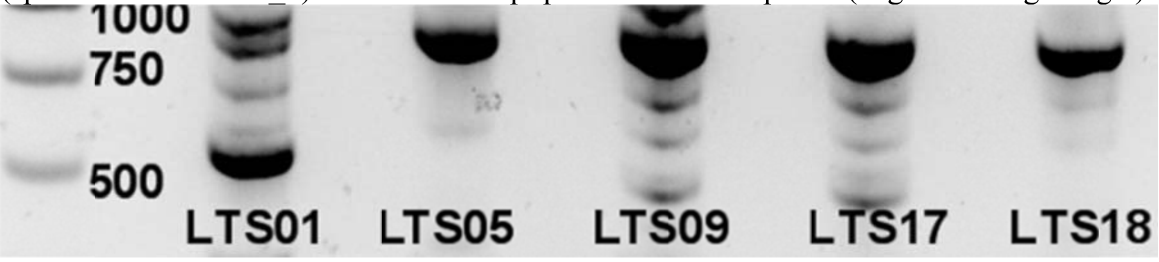

The founder plant in lane 1 has three major bands which all appear to be single alleles and all BLAST as 6G-FFT_1. The remaining founders have a single major band comprising two alleles as distinguished by SNPs. The lower band in lane 1 is classified as a duplicate locus when it occurs in the GRASP progenies.

E. Example of banding pattern for primers LBTisopu6G-FFT F2.1 and LBTisopu6G-FFT R2 (specific for 6G-FFT_2) in F2 mapping family plants (negative image of gel)

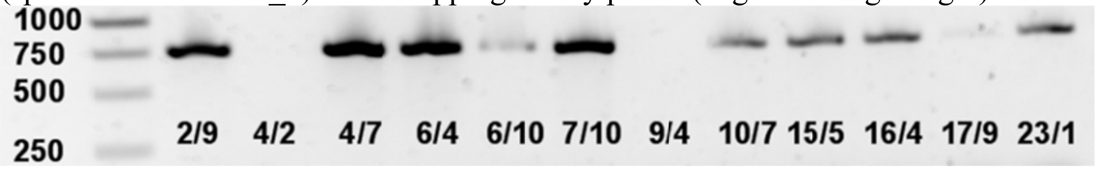

The single dominant band represents one allele at a second 6G-FFT locus absent in lanes 2 and 7

Figure S3. Mean seasonal carbohydrate content (mg/g DM) for A. The extreme low (L) and high (H) groups from the WSC F2 mapping family (n=12) over the growing season from April to November and for B. The low and high GRASP C2 populations (n=30) over the growing season from March to November. Polymeric fructan, black solid blocks; oligomeric fructan, grey solid blocks; disaccharides, hatched blocks; monosaccharides, open blocks. Total WSC, whole bars. Interaction LSD (0.05) where applicable is indicted by inset bars (total WSC is on the right)

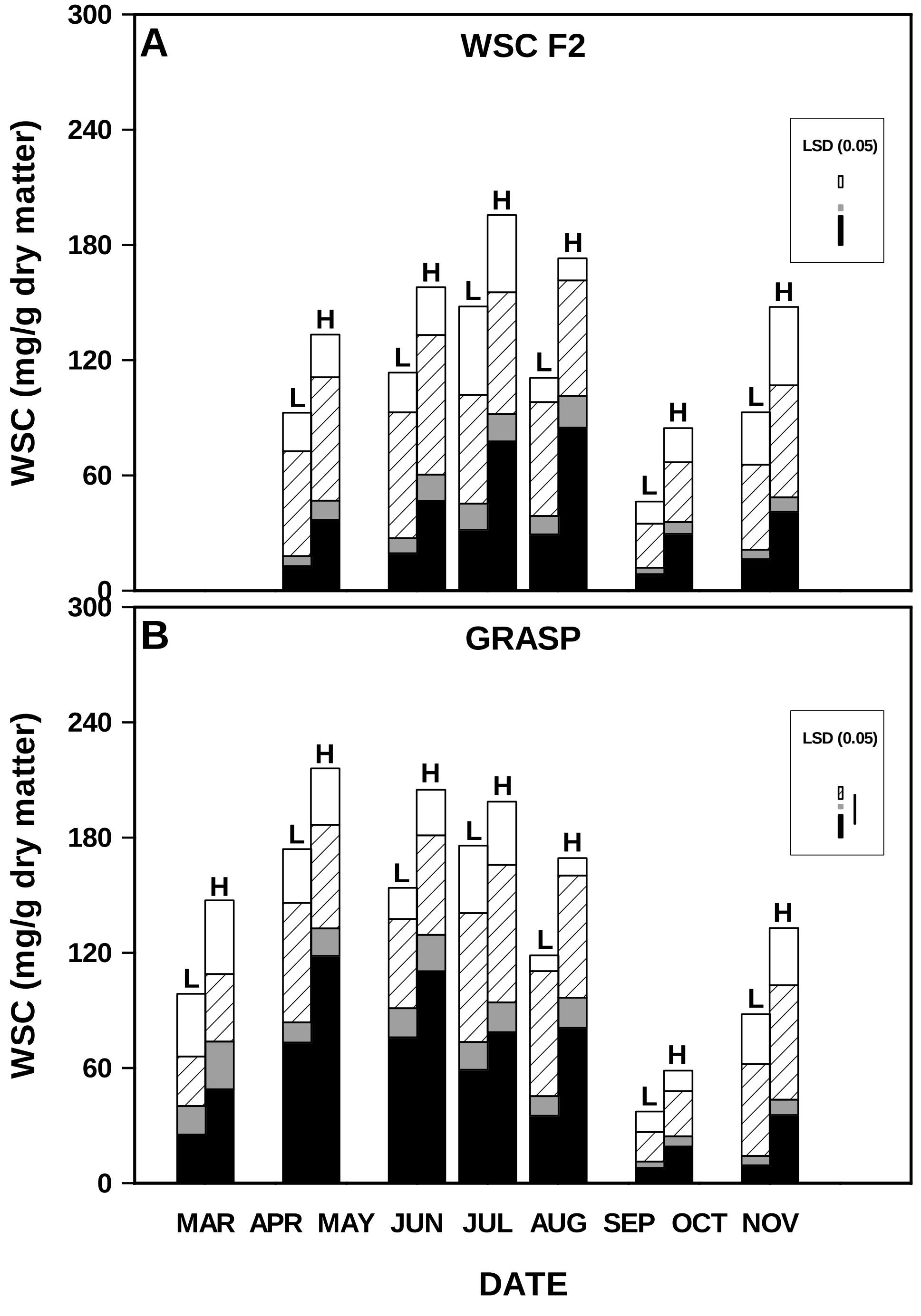

Supplement: Supplementary file 1 [file Presentation1.PDF]
